# Substrate-specific pressure-dependence of microbial sulfate reduction in deep-sea cold seep sediments of the Japan Trench

**DOI:** 10.3389/fmicb.2012.00253

**Published:** 2012-07-17

**Authors:** Antje Vossmeyer, Christian Deusner, Chiaki Kato, Fumio Inagaki, Timothy G. Ferdelman

**Affiliations:** ^1^Department of Biogeochemistry, Max Planck Institute for Marine MicrobiologyBremen, Germany; ^2^GEOMAR Helmholtz Centre for Ocean ResearchKiel, Germany; ^3^Marine Ecosystems Research, Japan Agency for Marine-Earth Science and TechnologyYokosuka, Japan; ^4^Kochi Institute for Core Sample Research, Japan Agency for Marine-Earth Science and TechnologyNankoku, Kochi, Japan; ^5^Submarine Resources Research Project, Japan Agency for Marine-Earth Science and TechnologyNankoku, Kochi, Japan

**Keywords:** hydrostatic pressure, deep sea, sulfate reduction, piezophile, biogeochemical processes

## Abstract

The influence of hydrostatic pressure on microbial sulfate reduction (SR) was studied using sediments obtained at cold seep sites from 5500 to 6200 m water depth of the Japan Trench. Sediment samples were stored under anoxic conditions for 17 months in slurries at 4°C and at *in situ* pressure (50 MPa), at atmospheric pressure (0.1 MPa), or under methanic conditions with a methane partial pressure of 0.2 MPa. Samples without methane amendment stored at *in situ* pressure retained higher levels of sulfate reducing activity than samples stored at 0.1 MPa. Piezophilic SR showed distinct substrate specificity after hydrogen and acetate addition. SR activity in samples stored under methanic conditions was one order of magnitude higher than in non-amended samples. Methanic samples stored under low hydrostatic pressure exhibited no increased SR activity at high pressure even with the amendment of methane. These new insights into the effects of pressure on substrate specific sulfate reducing activity in anaerobic environmental samples indicate that hydrostatic pressure must be considered to be a relevant parameter in ecological studies of anaerobic deep-sea microbial processes and long-term storage of environmental samples.

## Introduction

Sediments that cover the deep seafloor are typically marked by cold temperatures of less than 2°C and high hydrostatic pressures greater than the average ocean pressure of 38 MPa (Jannasch and Taylor, 1984). Biogeochemical processes occurring during early diagenesis of such deep marine sediments contribute significantly to element cycling on a global scale when considering that the deep-sea comprises 75% of the total ocean's volume. A number of these processes are catalyzed by the combined activity of heterogeneous associations of microorganisms. Many prokaryotes in deep-sea sediments are adapted to the prevailing cold and high-pressure conditions, and they can be classified as psychrophiles or piezophiles, respectively (Kato, 1999). Like psychrophiles, whose mechanisms to cold adaptation include the integration of higher amounts of polyunsaturated fatty acids into the membrane at decreasing temperature (Yayanos et al., 1982), distinct physiological mechanisms of adaptation to high hydrostatic pressure exist (Yayanos et al., 1982; DeLong and Yayanos, 1986; Bartlett et al., 1989; Chastain and Yayanos, 1991; Mozhaev et al., 1996; Kato, 1999; Li et al., 1999). Changes in the structure of the cell membrane, alterations in protein function within in the respiratory chain, and variations in enzyme activity are typically observed characteristics of adaptation to high hydrostatic pressure (DeLong and Yayanos, 1985; Gross and Jaenicke, 1994; Yayanos, 1995; Abe et al., 1999; Boetius and Lochte, 2000; Macgregor, 2002). These different cellular adaptation mechanisms to temperature and pressure may be expressed at the population level as changes in biogeochemical process rates. Nevertheless, as compared to the effects of temperature, little is known about the ecologically relevant effects of hydrostatic pressure and changing pressure on biogeochemical processes, although half of all prokaryotes live in high-pressure environments (Whitman et al., 1998). It remains to be established how sensitive microbial communities in the deep-sea are to hydrostatic pressure.

Most previous studies have focused on aerobic microorganisms, and therefore do not provide insight into the effects of pressure on turnover rates in the deep-sea sediments where the dominant pathways for the mineralization of organic matter are carried out by anaerobic organisms. Contrary to aerobic mineralization, a chain of microbial communities act in concert during the anaerobic degradation or organic carbon. A pressure effect on the overall anaerobic process rates is hard to predict from pure culture studies alone. Furthermore, the effect of high pressure and pressure change on aerobic process rates in the benthic layers of deep-sea sediments might be more pronounced than on anaerobic process rates, because the aerobic response may show a mixed signal from autochthonous piezophilic bacteria in the sediment as well as piezotolerant organisms from the water column. Pressure tolerant organisms can be transported by faecal pellets or other fast sinking particles (i.e., marine snow) from surface waters to the deep-sea over the depth of 4500 m in 4–6 weeks (Abe et al., 1999) and may remain dormant or inactive cells on the seafloor (ZoBell and Morita, 1957). These fast-sinking particles, however, probably do not harbor the anaerobic communities that would be characteristic of sub-seafloor microbial communities. Only deeply buried, deep-sea sediments will presumably favor the establishment and maintenance of a pressure-adapted autochthonous community of anaerobic organisms. Consequently, biogeochemical and ecological studies addressing the influences of hydrostatic pressure on anaerobic systems must consider additional factors including the nature of the terminal electron accepting process, substrate availability and population adaptation time.

Microbially mediated sulfate reduction (SR), as represented in Equation 1, is a major terminal electron accepting process in the degradation of organic matter in marine sediments (Equation 1) (Jørgensen, 1982; Thamdrup and Canfield, 1996; Ferdelman et al., 1997, 1999; D'Hondt et al., 2004).

(1) $$2{\text{CH}}_{2}\text{O}+{\text{SO}}_{4}^{2-}\to 2{\text{HCO}}_{3}^{-}+{\text{H}}_{2}\text{S}$$

**Figure 1:**
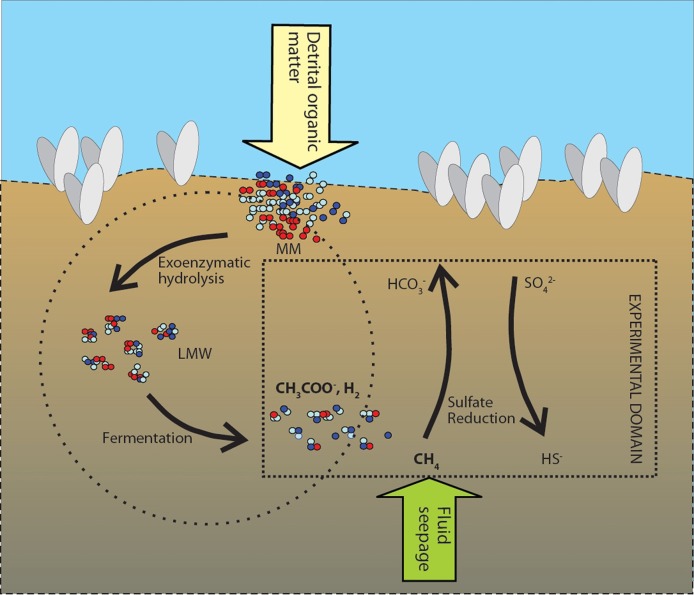
**Model of the anaerobic food web coupled to sulfate as the terminal electron accepting process (after Jørgensen, 2006) for the Japan Trench sediments.** Detrital organic matter is buried in the surface sediment as macromolecules (MM). During exoenzymatic hydrolysis macromolecules are broken down to low molecular weight molecules (LMW). Fermentation further degrades LMW to short chain fatty acids such as acetate, and hydrogen. Fluid seepage delivers additional methane to the near-surface sulfate-bearing sediments. Calyptogena colonies also thrive on methane seepage and chemosynthetic sulphide reducers. In addition to pressure, we manipulated concentrations of substrate methane, acetate and hydrogen (marked in bold) in order to tease out effects of pressure on the sulfate reducing community.

It is a key process in the coupling of the carbon and sulfur biogeochemical cycles. Moreover, SR serves as an excellent model for investigating the effects of pressure and temperature on biogeochemical processes in marine sediments, as it is a terminal step in the overall process of the anaerobic breakdown of detrital organic matter. In experiments using radio-labeled ^35^S-sulfate to trace rates of SR, we in fact measure the rate at which the terminal oxidation step proceeds, whereby fermentation products such as H_2_, short-chain carboxylic acids, and methane, among other products, are oxidized using sulfate (Figure 1). As such, the terminal sulfate reducing step depends on a series of exoenzymatic hydrolyses and fermentation reactions. We can, therefore, link quantitatively the rate of SR with the overall turnover of detrital organic carbon (the CH_2_O in Equation 1) buried in the sediments. Any pressure effect on any step during the anaerobic degradation of organic carbon in Figure 1, whether positive or negative, will have a corresponding effect on the terminal sulfate reducing step.

In addition to organoclastic SR associated with the degradation of buried organic matter (Equation 1), external substrate supply in the form of methane may fuel SR via methane-dependent SR (or anaerobic oxidation of methane, AOM).

(2) $${\text{CH}}_{4}+{\text{SO}}_{4}^{2-}\to {\text{HCO}}_{3}^{-}+{\text{HS}}^{-}+{\text{H}}_{2}\text{O}$$

Fluids emanating from cold-seeps associated with faults, pockmarks and mud volcanos are often rich in methane supplied from deep reservoirs. In such systems, consortia of archaea, for instance ANME, and delta proteobacteria associated with sulfate reducing bacteria, act to oxidze methane and reduce sulfate. As shown in Figure 1, these AOM communities are fed directly with substrate independent of the enzymatic and fermentative pathways associated with organoclastic SR.

In spite of the numerous studies of marine SR and AOM, only few data are available on the effects of high hydrostatic pressure on anaerobic biogeochemical processes including microbial SR. ZoBell and Oppenheimer (1950), however, observed enhanced sulfide production at *in situ* pressure. Kallmeyer and Boetius (2004) determined SR rates and rates of anaerobic methane oxidation (Equation 2) in sediments from hydrothermal sediments in the Guaymas Basin at different pressures and temperatures. They demonstrated that SR rates were highest at 45 MPa at temperatures of 95°C, which is *in situ* temperature, however, double the *in situ* pressure. Bowles et al. (2011a) demonstrated on cold seep samples that at elevated pressure and elevated concentrations of methane, rates of methanogenesis, anaerobic methane oxidation and SR increased compared to atmospheric pressure incubations.

In this study, we investigated the effect of hydrostatic pressure on microbial SR in sediment samples obtained from two cold seep-sites at the slope of the Japan Trench. Samples were taken at the rim of colonies close to the world's deepest *Calyptogena phaseoliformis* colonies (Fujikura et al., 1999; Li et al., 1999). Terminal restriction length polymorphism (T-RFLP) analysis of 16S rRNA genes amplified from two stations from in- and outside the *Calyptogena* colonies was conducted as a fingerprint method for characterization of the microbial community. SR was compared in incubations at 0.1 and 50 MPa starting from samples stored at *in situ* pressure, at atmospheric pressure or under methanic conditions with a methane partial pressure of 0.1 MPa. Pressure regulation of SR was further studied after amendment with acetate, hydrogen or methane as putative site-specific electron donors for microbial SR.

## Materials and methods

### Sample site and collection

**Figure 2:**
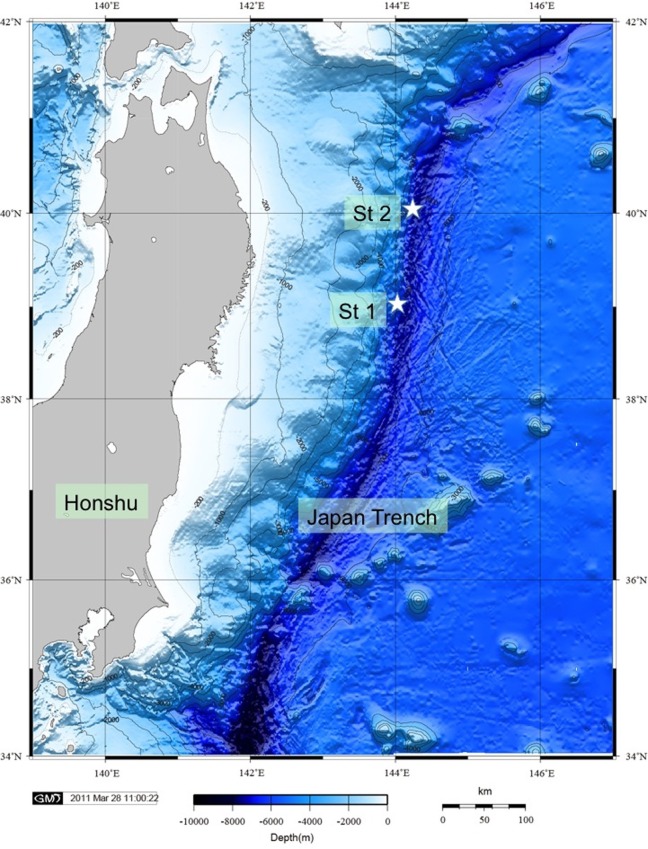
**Bathymetric map of the Japan Trench showing the positions of Station 1 and 2**.

Deep-sea cold seep sediment samples from two stations at the landward slope of the Japan Trench (Fujikura et al., 1999) were obtained with a manned submersible *Shinkai 6500* during YK 06–05 cruise of the *R/V Yokosuka* in May 2006 (Figure 2). The Japan Trench is associated with the Northeast Japan Arc, which hosts the island of Honshu. This area experienced nine earthquakes of magnitude 7 or greater since 1973, according to the U.S. Geological Survey (USGS). The most recent and strongest event with magnitude 8.9 was observed in March 2011. Cold seeps, which are caused by the convergent subduction of the Pacific Plate under the North American Plate, have been observed along faults in the deep Japan Trench (Li et al., 1999), These faults create pathways for reduced compounds to percolate and seep through rock and sediment to the sediment-water interface where reduced compounds may fuel chemosynthetic communities (see below).

Station 1 is located on a steep slope in a water depth of 6177 m at 40°6.025′N, 144°53.562′E. Station 2 lies on a relative plain in 5347 m water depth at 39°6.356′N, 143°53.562′E. Sand and mudstones characterize the Station 1 sediment facies, while Station 2 sediments are composed of mud, pelagic clay and mud mixed with stones. At both stations, the chemistry of the sediment supports a habitat for chemosynthetic clam colonies of *Calyptogena phasaeoliformis*. Porewater profiles of sulfate, dissolved inorganic carbon and ammonium concentrations obtained during the YK 06-05 cruise indicated, however, that methane seepage was much more active and closer to the surface at Station 2. Sulfate concentrations at Station 2 were depleted within 20 cm of the surface at sites within the *Calyptogena* colonies. For SR experiments in this study, we used sediments from dive 6K-952, Station S1, and dive 6K-955, Station S2. Samples for molecular studies were obtained from dives 6K-950, −953, at Station 1 and dives −954, −955, and −957, Station 2 (Table 1).

**Table 1:** **Samples used in this study: sampling station, water and sediment depth, and position relative to the Calyptogena colony (n.s.: not specified)**.

**Dive number**	**Station**	**Sample ID**	**Depth [m]**	**Sediment depth [cm]**	**Position relative to clam colonies**
950	1	6K950 s	6177	0–5	adjacent
952	1	6K952	6177	n.s.	n.s.
953	1	6K953	6265	n.s.	inside
954	1	6K954	6265	n.s.	adjacent
955	2	6K955 s	5347	0–5	inside
955	2	6K955 m	5347	10–15	inside
957	2	6K957 s	5347	0–5	inside
957	2	6K957 m	5347	10–15	inside

### Onboard preparation of slurries for storage

All samples experienced slow decompression during recovery from the seafloor. The decompression rate was approximately 0.4 MPa/min (42 m/min) resulting from the rise velocity of the submersible. After recovery onboard the support vessel YOKOSUKA sediment from subcores was processed within 1–4 hours. Sediment aliquots obtained from pushcore samples were diluted 1:5 with anoxic sulfate reducing bacteria medium (SRB medium) (0.756 mM KBr, 8.05 mM KCl, 10 mM CaCl_2_^*^2H_2_O, 27.89 mM MgCl_2_^*^6H_2_O, 27.6 mM MgSO_4_^*^7H_2_O, 451 mM NaCl). The slurry was transferred to serum bottles or Hungate tubes that were subsequently closed with butyl septa and aluminum crimps. The samples were transported and stored under anoxic conditions at 4°C and 0.1 MPa (S1, S2) or 50 MPa (S1-50, S2-50) in high pressure stainless steel autoclaves with a maximum pressure capacity of 50 MPa. For samples stored under high-pressure conditions, 3 ml Luer Lok™ plastic syringes partially filled with media were connected to the slurries via a 0.9^*^40 mm Erosa™ needle inserted through the septum to compensate for pressure changes during re- and decompression (Nauhaus et al., 2002). For storage under methanic conditions, sediment from subcores was pushed into 1 l DURAN® bottles prepared with anoxic SRB medium with an approximate dilution of 1:5. Sample bottles were closed immediately with butyl rubber septa. Headspace was exchanged through continuous flushing with methane for at least 10 min and samples were transported and stored under a large methane headspace (approximately 0.5 l and 0.1 MPa overpressure) at 4°C (total pressure 0.2 MPa). Headspace pressure was controlled prior to incubation experiments and SR activity was monitored on a regular basis via sulfide measurements. For incubations we chose slurries where sulfide production appeared to be highest (data not shown).

### Incubation experiments

After 17 months of storage as described above, samples and slurries for the incubation experiments were prepared in an anoxic chamber with N_2_/CO_2_ (90/10) atmosphere. For the incubation experiments, 5 ml Hungate tubes were filled with 1 ml of sediment slurry; 4 ml of SRB medium was added either without substrate amendment or after addition of H_2_ (1 mM), CH_3_COO^−^ (0.2 mM) or CH_4_ (1 mM); and then 100 μl of ^35^SO_4_^2−^(300 kBq ml^−1^) was added to each batch. The medium was also prepared with Resazurin as a redox indicator. The headspace-free incubations were carried out in quintiplicate. Hydrostatic pressure was applied using an Enerpac® (M-1000) multi-fluid hand pump with a maximum working pressure of 100 MPa. Incubations were performed at 4°C for 29 days at either 50 MPa in high-pressure autoclaves or at 0.1 MPa.

After the incubation, SR activity was stopped by mixing the slurry into 5 ml of a 200 g/l zinc acetate solution. Measurements of SR were performed using the single step cold chromium distillation (Kallmeyer et al., 2004). The SR rate is calculated and reported as the amount of sulfate reduced over an incubation time of 29 days per gram dry weight of the sediment slurry (nmol·gdw^−1^). SR reduction blanks on sediment samples were determined on killed samples. At the beginning of the experiment slurry and tracer were directly added to the zinc acetate solution and frozen. Reduced sulfide was determined as above for the other samples. It should be noted that blank values were determined on slurries prepared from both Stations 1 and 2, and on sediments stored with and without pressure, and stored under methanic conditions. However, we did not run killed controls for the incubation experiments under high pressure conditions. A detection limit of 11 nmol gdw^−1^ was determined according to Kallmeyer et al. (2004) on pooled determination of all blank values (*n* = 58).

Comparison between medians of separate experiments were made with the non-parametric two-tailed Mann-Whitney U test (*n* = 5 for each sample set) using the software package PAST (Hammer et al., 2001).

### DNA extraction and T-RFLP analysis of 16s rRNA genes

For the isolation of environmental DNA, samples from dives 6K-950, −953 and −954 from Station 1 and 6K-955 and −957 from Station 2 at the different sediment depths (see Table 1) were frozen immediately (−80°C) on board and returned to the Japan Agency for Marine Earth Science and Technology (JAMSTEC). Total DNA was extracted directly from the sediment samples using Ultra Clean Soil DNA Kit (MO Bio Laboratories, Solana Beach, CA, USA). Bacterial and archaeal 16S rRNA genes were amplified by the polymerase chain reaction (PCR) with domain Bacteria- and Archaea-specific primer sets as described previously (Kato et al., 1997). The fragments of bacterial and archaeal 16S rRNA genes for T-RFLP analysis were amplified by PCR using a primer set of Bac27F and FAM-labeled Bac927R (Inagaki et al., 2002), and Arch21F and FAM-labeled Arch958R (Inagaki et al., 2001), respectively. Amplified products were purified using a Gel Spin DNA purification kit (MO Bio Laboratories). The purified 16S rRNA gene fragments were digested with *Hha*I at 37°C for 8 h. The electrophoretic patterns of terminal restriction fragments (T-RFs) were analyzed using a model 3130 automated sequencer (Perkin-Elmer/Applied Biosystems) equipped with GENESCAN software ver. 3.1 according to the manufacturer's protocol (Perkin-Elmer/Applied Biosystems). The precise lengths of the T-RFs were determined by comparison with an internal size standard (GENESCAN-2500 ROX, Perkin-Elmer/Applied Biosystems) added to each digested sample. Phylogenetic position of the major T-RF peaks was assigned based on 16S rRNA gene sequences that have been obtained by the clone library analyses from cold seep sediments in the Japan Trench (Inagaki et al., 2002; Kato et al., 2008) and other locations (e.g., Knittel et al., 2005; Loesekann et al., 2007).

## Results

### Molecular characterization of deep-sea cold-seep microbial communities

**Figure 3:**
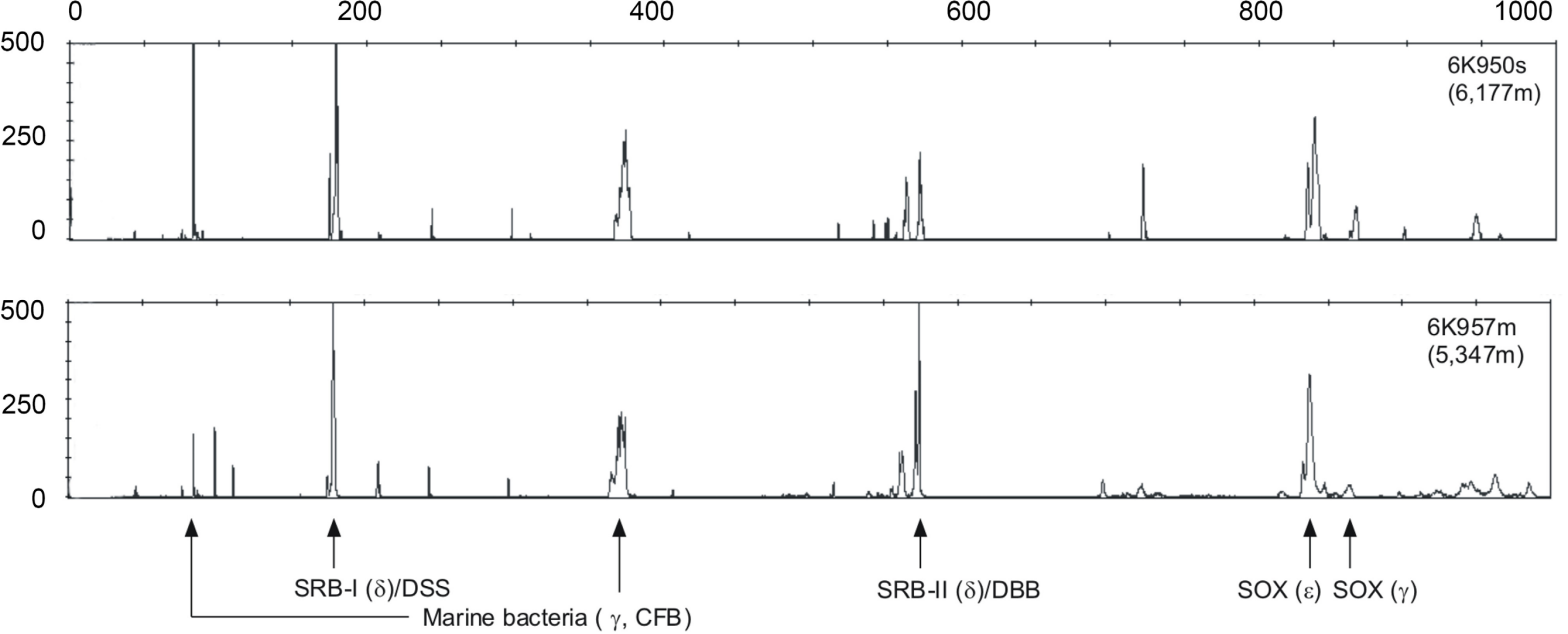
**Examples of bacterial T-RFLP electrophoretograms from Station 1 (upper profile) and Station 2 (lower profile).** γ, δ and ε indicate the corresponding proteobacterial groups, and Sox and SRB indicate the sulfide oxidizing and sulfate reducing bacterial groups. The lengths of the fragments are displayed on the x-axis and relative fluorescence intensity of peaks is shown on the y-axis.

Based on the fluorescence integrity of fragment lengths (T-RFs), γ-, ε-, and δ-proteobacteria were all detected in the bacterial T-RFLP profiles (Figure 3), consistent with earlier studies (Inagaki et al., 2002; Kato et al., 2008). Given the potential PCR biases and the non-quantitative nature of this profiling method, the electrophoretograms from both stations are strikingly similar. The only slight exception is that Station 1 appears to have a more dominate cluster in the γ-Marine Bacteria group as compared to Station 2. The δ-proteobacteria were dominated by peak intensities in the DSS (*Desulfosarcina-Desulfococcus*) branch (Knittel et al., 2005) and the DBB (*Desulfobulbus*) branch (Loesekann et al., 2007), whereas ε-proteobacteria (Sox) related to the genus *Sulfurimonas* (Inagaki et al., 2003) dominated peaks.

**Figure 4:**
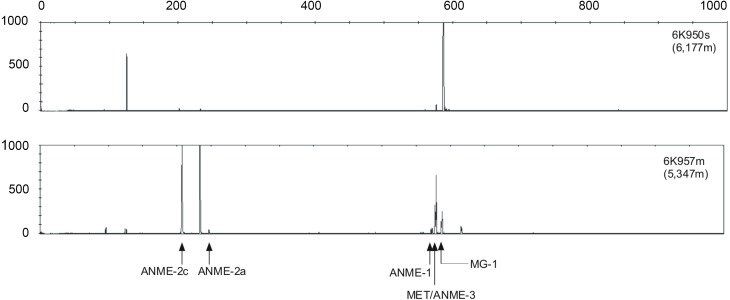
**Examples of archaeal T-RFLP electrophoretograms from Station 1 (upper profile) and Station 2 (lower profile).** MG-I, MET and ANME indicate the crenarchaeota marine group I, the methanogenic euryarchaeota group, and the anoxic methane oxidizing archaea group, respectively. The lengths of the fragments are displayed on the x-axis and relative fluorescence intensity of peaks is shown on the y-axis.

T-RFLP fingerprinting of archaeal 16S rRNA genes revealed differences in community structure between Station 1 and Station 2 (Figure 4). At Station 2, mainly ANME-2a, ANME-2c and ANME-3 (and some methanogenic archaea) groups were predominantly detected in the sediment samples. Fluorscence intensity at Station 1, in contrast, was dominated by peaks associated with the Marine Crenarchaeota Group I (MG-I), although small peaks of ANME-2a, ANME-2c and ANME-3 were also detected.

### Sulfate reduction in sediments stored under organoclastic conditions

**Figure 5:**
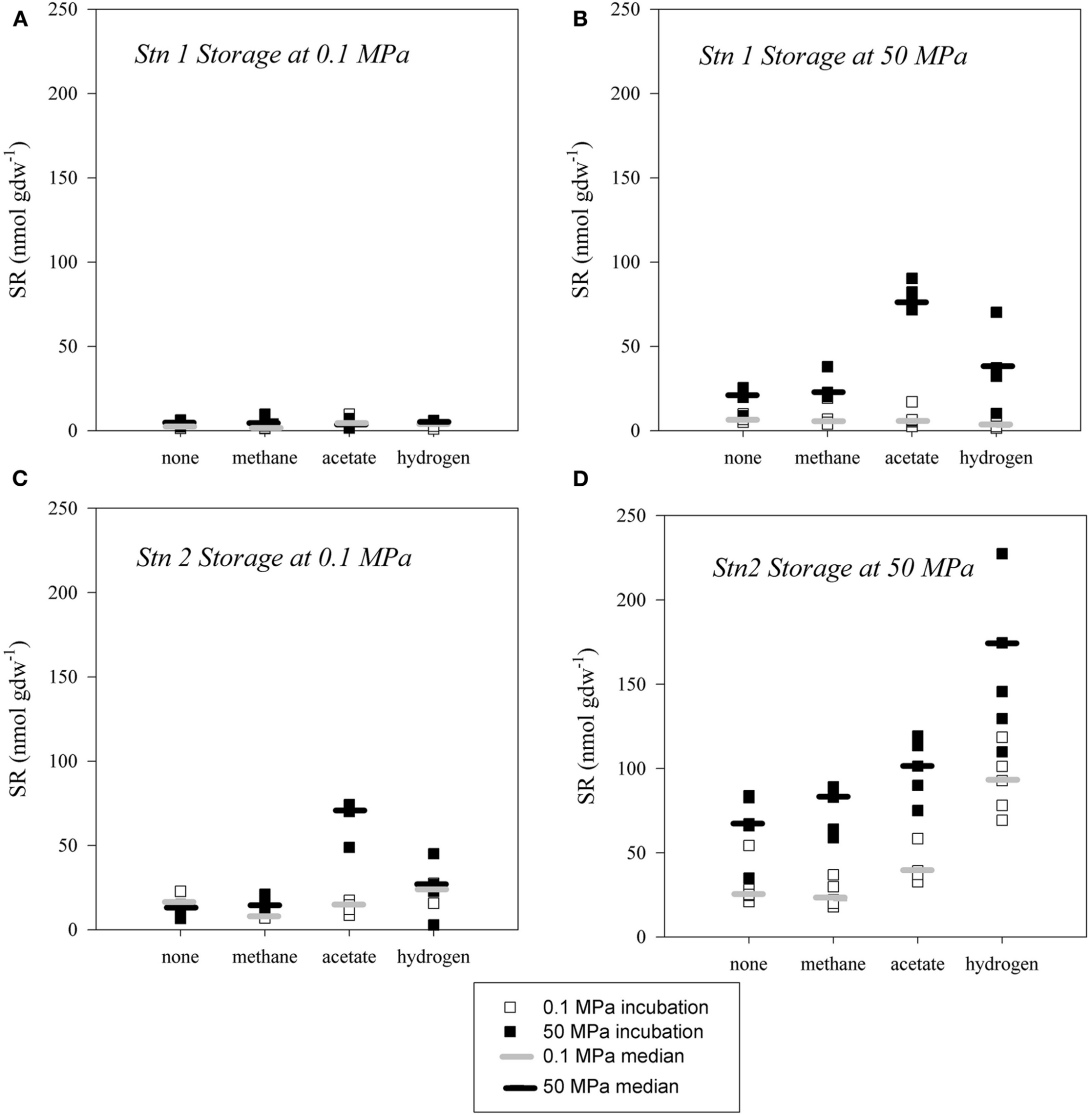
**Extent of sulfate reduction over 29 days in sediment slurries from Station 1 (A,B) and Station 2 (C,D), stored at atmospheric pressure (A,C) and at *in situ* pressure (B,D) under organoclastic conditions, respectively.** Incubations were conducted at atmospheric pressure (hollow square, median denoted as light grey horizontal bar) and high pressure (filled square, median denoted as black horizontal bar) and at 4°C, without addition of substrate (none) or amended with methane, acetate or hydrogen.

In sediment samples from Japan Trench stored under organoclastic sulfate reducing conditions SR was only detected in the samples stored and incubated at *in situ* pressure (i.e., 50 MPa; Figures 5B,D). Samples that remained at atmospheric pressure after retrieval from the seafloor and during storage did not show any detectable sulfate reducing activity (<11 nmol gdw^−1^; Figures 5A,C). In all samples from Station 1 (Figure 5A), sulfate turnover was neither stimulated by pressure application nor substrate addition. The only exceptions were the acetate and hydrogen amended samples from Station 2 stored at 0.1 MPa, which exhibited enhanced activity when incubated at 50 MPa.

In contrast, sediments stored under *in situ* pressure and amended with substrate during incubation experiments also exhibited a positive effect when incubated under pressure (*p* < 0.05). In sediment from Station 1 stored at *in situ* pressure (Figure 5B), the highest yields of SR were detected in high-pressure incubations in acetate amended samples (70–80 nmol gdw^−1^) and with addition of hydrogen (30–45 nmol gdw^−1^). Upon methane addition SR at 50 MPa was only slightly elevated (20–38 nmol gdw^−1^). In comparison, incubation experiments at 0.1 MPa with either substrate (acetate [2–17 nmol gdw^−1^], hydrogen [1–5 nmol gdw^−1^] and methane [3–19 nmol gdw^−1^]) amendments were close or below the limit of detection [<11 nmol gdw^−1^]. The greatest extents of SR were observed in the hydrogen amended batches at 50 MPa (110–230 nmol gdw^−1^) from Station 2. These were on average about 30% higher than rates in 0.1 MPa incubations (70–120 nmol gdw^−1^). Overall, the samples from Station 2 that were stored at *in situ* pressure of 50 MPa and then incubated at 50 MPa were substantially more active than the corresponding samples incubated at 0.1 MPa (Figure 5D) or stored at 0.1 MPa (Figure 5C).

### Sulfate reduction in sediment stored under methanic conditions

**Figure 6:**
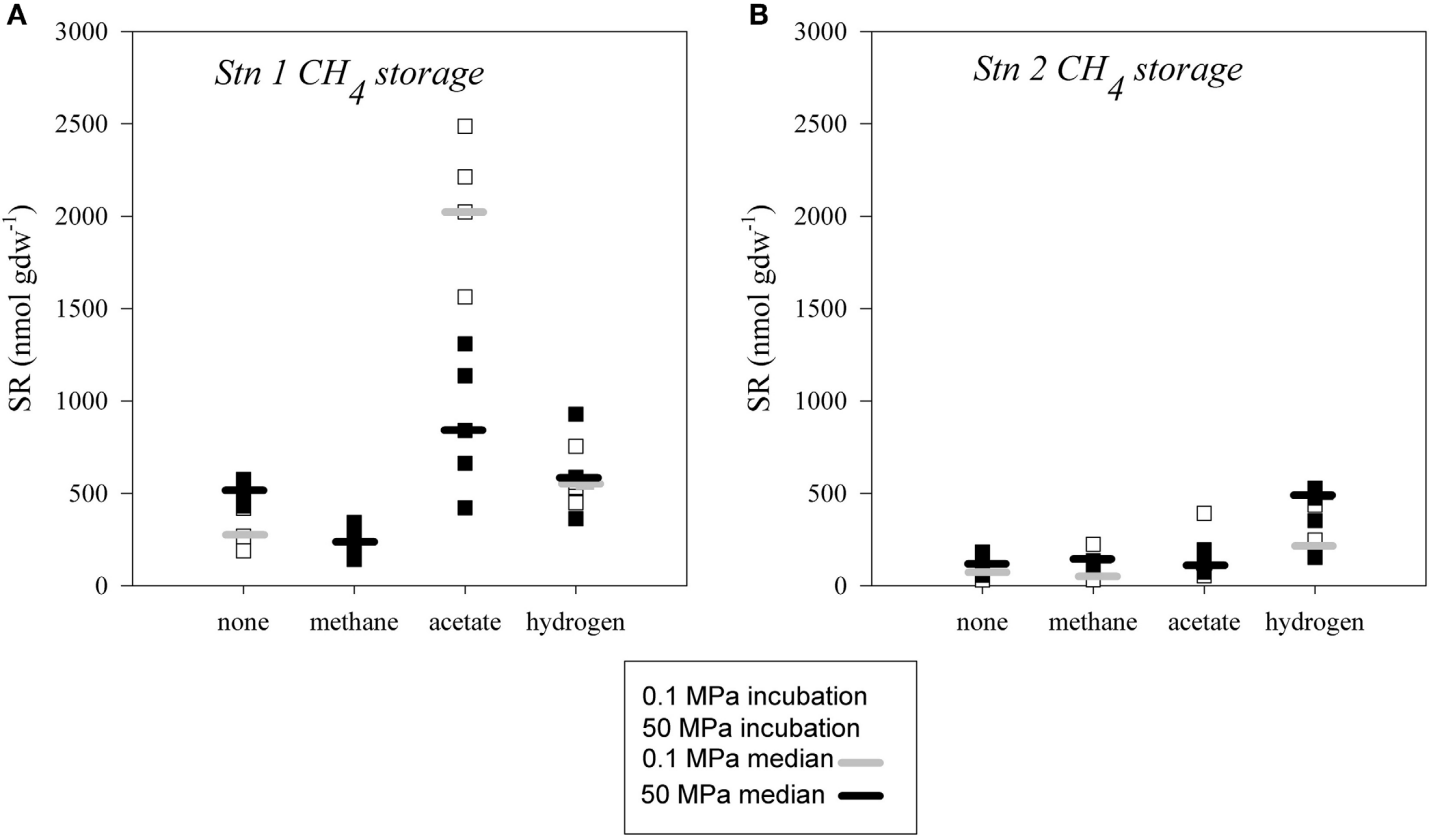
**Extents of sulfate reduction in sediment slurries stored with a methane partial pressure of 0.2 MPa at (A) Station 1 and (B) Station 2.** Incubations were conducted at atmospheric pressure (hollow square, median denoted as light grey bar) and *in situ* pressure (filled square, median denoted as black bar) and at 4°C, without addition of substrate (none) or amended with methane, acetate or hydrogen.

Sulfate reduction in slurries incubated after seventeen months of storage under methanic conditions (0.1 MPa methane overpressure; 0.2 MPa total pressure) were up to one order of magnitude higher than in samples stored under organoclastic sulfate reducing conditions even without the addition of a substrate during incubation (Figure 6). A difference between atmospheric and *in situ* pressure incubations without amendment could only be observed at Station 1, where SR at high pressure exceeded that at atmospheric pressure (Figure 6A). In constast, the extent of SR was greatest upon acetate amendment (1100–2500 nmol gdw^−1^) at atmospheric pressure at Station 1 (Figure 6A). Recompression to 50 MPa appeared to depress the acetate supported SR rate substantially. Rates of SR tended to be elevated with the addition of hydrogen, but on average there was no difference in activity between the different pressure incubations (350–930 nmol gdw^−1^ at Station 1 and 150–530 nmol gdw^−1^ at Station 2).

## Discussion

### A piezophilic sulfate-reducing community in the japan trench?

The incubation experiments clearly indicate that the anaerobic prokaryotic community found in and around the cold seep sediments at great depths of Japan Trench consists of sulfate reducing organisms adapted to the high pressure conditions of >50 MPa. The prokaryotic communities in non-substrate amended sediment slurries from 5347 to 6177 m water depth remained more active after 17 months of storage at *in situ* pressure than those under low-pressure storage. Moreover, rates of SR remained elevated only when high-pressure conditions were maintained. A decrease of pressure to atmospheric levels (0.1 MPa) resulted in the loss of activity in both substrate-amended and non-amended experiments. Elevated activity at *in situ* pressure (up to 30% in samples from both Station 1 and Station 2 stored at 50 MPa, Figures 5B,D) was consistent with earlier unpublished observations from deep sediments (5400 m water depth) of the Peru Margin where rates of SR doubled with increased pressure (Parkes and Ferdelman, unpublished data).

The enhancement of sulfate reducing activity with the addition of substrate in the samples stored under *in situ* pressure conditions indicates that the sulfate reducing microbes themselves are piezophiles. We decoupled the effects of pressure on enzymatic hydrolysis and fermentation from SR by adding non-limiting concentrations of substrates for SR (as per Isaksen and Jørgensen, 1996). Fermentation products, such as hydrogen and acetate are normally maintained at micromolar to sub-micromolar concentrations by sulfate reducing communities (Finke and Jørgensen, 2008; Figure 1). As shown in Figures 5B,D, the addition of excess amounts of fermentation substrates acetate, hydrogen or methane, led to an increase in the rates of SR in those samples that have been stored at in situ pressure and that were further incubated at high pressure. Thus, a fraction of sulfate reducing prokaryote community in our experiments was directly sensitive to pressure.

Acetate-oxidizing sulfate reducers appeared to be the most strongly affected by pressure, even in samples that were stored at atmospheric pressure. In deep cold seep sites of the Japan Trench, which are the world's deepest *Calyptogena phaseoliformis* colonies (Li et al., 1999), SR may be penultimately fueled by bivalve biomass subject to degradation by fermenting organisms that presumably produce short chain fatty acids and/or hydrogen. The addition of small amounts of these putative substrates stimulated SR considerably in high-pressure incubations and thus is indicative of their relevance *in situ*. From the specific response to acetate or hydrogen, we assume that the overall higher activity observed in sediments stored at high pressure was not necessarily due to piezophily within the fermenting community, but could be attributed to piezophilic sulfate reducing bacteria.

As yet, only two piezophilic sulfate reducing bacteria have been isolated. They belong to the branch *Desulfovibrio* (Bale et al., 1997; Alazard et al., 2003) in the δ-Proteobacteria, whereas most other piezophilic isolates belong to the γ-Proteobacteria (Kato et al., 1995, 1997; DeLong et al., 1997; Nogi and Kato, 1999; Nogi et al., 2002; Xu et al., 2003). The sulfate reducers in our sample are perhaps related to the DBB or DSS branches of δ-Proteobacteria. Earlier T-RFLP studies at Station 1 (Inagaki et al., 2002) also revealed δ- and ε-Proteobacteria, including sequences of *Desulfosarcina.* Sulfate reducers are, therefore likely to be a key pieziophilic component of these deep-sea seep sites.

The reduced SR activity in samples stored at 0.1 MPa prior to incubation experiments (Figures 5A,C), with the exception of the acetate amendment in Station 2 sediments (Figure 5C) indicated a partial loss of viability of the pressure sensitive prokaryotic community. It is not entirely possible, however, to distinguish whether the loss in catabolic activity resulted from inhibition of sulfate reducing organisms or from organisms in partaking in the hydrolysis and fermentation steps. If the latter was true, the decrease in SR activity in samples stored at 0.1 MPa could also result from starvation of the sulfate reducing community during storage. The effect of pressure on the fermentative prokaryotic community remains to be explored.

### Methane and the deep japan trench sulfate reducing communities

The association of microorganisms with the anaerobic oxidation of methane oxidation at the deep Japan Trench sites was evident in the vastly enhanced SR activity increase after storage under methanic conditions, and was consistent with the presence of ANME sequences in most seep sediment samples inside the *Calyptogena phaseoliformis* colonies. Thus, seepage of methane ultimately provides the source of electron donor for SRB (Masuzawa et al., 1992). The clear fingerprint of ANME-2c/DSS and ANME-3/DBB at Station 2 provides evidence that the methylotroph seep communities were still active at the time of sampling, which concurs with the findings of Arakawa et al. (2005). Likewise, Li et al. (1999) have shown that δ-Proteobacteria at similar sites in the Japan Trench are present and cluster mainly with sequences of sulfate or sulfur reducing bacteria (e.g., *Desulfosarcina variabilis*, *Desulfobacterium* sp. BG33). Delta-proteobacteria are important for sulfur cycling inside the *Calyptogena* colonies, reducing sulfate to sulfide and thus providing the energy source for sulfide oxidizing endosymbiotic bacteria of the bivalves (Li et al., 1999).

Interestingly, SR activity in samples stored long-term under methane, did not correlate with changing pressure. Overall, the rates of SR were much more enhanced in the samples stored with methane (Figure 6), but no clear pressure effect related strictly with methane could be ascertained in these experiments. The addition of extra methane had no effect on the SR rates, independent of whether pressure was applied or not. Considering that the sampling sites are influenced by active seepage and that the presence of anaerobic methanotrophs was confirmed by T-RFLP, it is surprising that methane did not appear to be a good substrate for sulfate reducers in short-term incubations. A similar effect was observed in a study by Bowles et al. (2011a), where methane addition at *in situ* pressure had positive effects on methanogenesis and AOM rate measurements, however, had very little effect on SR. Orcutt et al. (2004) and Bowles et al. (2011b) also observed that there is only a weak coupling between AOM and SR, with high sulfate reduction rates and very low AOM rates in sediments and seep sites in the Gulf of Mexico. Methane metabolism coupled to SR apparently differs from SR coupled to hydrogen or acetate oxidation (Boetius et al., 2000; Nauhaus et al., 2002). Several reaction steps are presumed to be thermodynamically unfavorable such as the enzymatic activation of the methane molecule as well as diffusion of a possible electron transfer compound between the organisms (Hoehler et al., 1994; Larowe et al., 2008; Thauer et al., 2008). In contrast to methane, hydrogen and acetate are readily converted by sulfate reducing bacteria. The positive effect of hydrogen and acetate addition in high-pressure incubations indicates the importance of various fermentation products as electron donors for the activity of sulfate reducing prokaryotes *in situ*.

### Storage and sampling effects

Almost all studies on the effect of pressure on microbiological and biogeochemical processes suffer from the unconstrained effects due to sample decompression during sample retrieval. We can not exclude the possibility that we have lost piezophilic activity simply by decompressing the samples during the initial sampling, and during the radio-tracer and substrate amendments during the high-pressure incubation set-up. (Psychophiles may have also suffered loss of activity and viability upon warming during the ascent of the submersible). The impact of mechanical stress during de- or recompression on prokaryotic cells in deep-sea sediment samples remains to be a matter of discussion. ZoBell and Morita (1957) described a high mortality rate of bacteria retrieved from deep-sea sediment. In tests with pure cultures, Yayanos et al. (1982) and Parkes et al. (1995) described cycles of de- and recompression that did not have an effect on metabolism of piezophilic bacteria although the total cell number decreased. A decompression of a few hours might not have a serious effect on piezophilic bacteria (Yanagibayashi et al., 1999) and only very rapid decompression seems to harm cells with gas vacuoles, as seen in studies with *Methanocaldococcus janaschii* (Park and Clark, 2002). In our experiments, we did not observe major changes in the overall activity after a few times of de- and recompressing as long as the samples were maintained at high pressure. Thus, a segment of the sulfate reducing community is not only piezophilic, i.e., its activity is greatest at the high *in situ* pressures typical for the deep Japan Trench, but can survive decompression and recompression.

In general, the comparison of sulfate turnover after different strategies of sample storage suggests that long-term decompression rather than short-term decompression leads to reduced microbial activity of microorganisms in deep-sea samples. Immediate e*x-situ* shipboard measurements of organoclastic SR in push cores at Station 2 were typically 2–5, but as high as 15 nmol cm^−3^ d^−1^ (J. Felden, pers. comm.). It is difficult to compare intact SR experiments with slurried sediments, but this would translate to 200–2500 nmol gdw^−1^ of SR under the conditions of our experiments. These rates were similar to the rates of SR measured in our samples stored under methane. Over longer periods of storage, however, this activity appeared to be lost, as was especially evident in the samples from Station 1 (Figure 5A) where bacteria did not show any activity after re-pressurization after storage at atmospheric pressure. They may have died or just lost their piezophilic character.

Loss of piezophilic character has been observed in the experiments of Parkes et al. (1995) using different pressures with sediment from the Peru Margin from 4500 m water depth, and where SR rates were greatest at atmospheric pressure. The samples from the Parkes et al. (1995) study were maintained for 6.5 years at atmospheric pressure and 4°C before incubation at 0.1, 45 and 90 MPa and 20°C. The microbial community reportedly had adapted to low pressure mesophilic temperatures. In contrast, some activity in Station 2 samples could be reconstituted after re-pressurization. It may be that the cells are dormant during long-term decompression and that this state is partly reversible when they are brought back to their habitat pressure. Such an effect is known in piezo-sensitive bacteria from low-pressure habitats that go into a state of suspended animation at high pressures (Abe et al., 1999).

Storage under methane had the greatest effect on the overall activity in our experiments. SR in samples stored under methanic conditions was much higher than in non-amended samples at atmospheric pressure. High concentrations of methane appeared to sustain high activities of a sulfate reducing community that was relatively less sensitive to pressure. It is interesting to note that the Station 1 samples stored under atmospheric conditions only exhibited a positive response to high pressure if they were stored under methane. It is even more striking that SR in the CH_4_ slurries from Station 1 strongly increased after acetate addition. We recognize that this suggests that acetate is an intermediate in AOM. However, current model assumptions and experimental data of the AOM mechanism exclude acetate as a specific intermediate in methane-dependent SR (Valentine and Reeburgh, 2000; Orcutt et al., 2008; Wegener et al., 2008). More likely, incubation under low-pressure and methanic conditions appears to have induced growth of an acetotrophic sulfate-reducing community in contrast to samples without methane addition. The enhanced SR rates in methanic experiments may simply reflect enhanced growth and therefore biomass production that had taken place earlier during storage. Whether the sulfate reducing community operating under methanic conditions was similar to the samples stored without methane remains to be tested; unfortunately, we do not have samples for this post-incubation analysis of a potential shift in the microbial community.

Recently, Mills et al. (2012) reported on community shifts after three months of storage at 4°C in a core from the Great Barrier Reef compared to shipboard samples. This core was taken in 167 m water depth and pressure may not be a critical storage requirement in shallower water. However, this reflects the importance of fixation of samples onboard to gain an impression of *in situ* microbial and geochemical parameters of deep sea sediments. Arakawa et al. (2006) observed extreme community changes in different pressure cultivations of sediments from the Japan Sea at over 3000 m water depth. The maintenance of strict anaerobic conditions during work with anoxic sediments and anaerobic processes is another important factor. Even the slightest oxygen penetration into the sample can lead to drastic community shifts (Lin et al., 2010). Our samples, however, were stored in glass, and did not show any indication of oxidation, as indicated by the use of a redox color indicator (i.e., Resazurin).

## Concluding remarks

Hydrostatic pressure does affect rates of SR in sulfate reducing communities associated with the *Calyptogena* colonies surrounding cold methane seep sites in 6000 m water depth in the Japan Trench. Our experiments show that the organoclastic sulfate reducing community is piezophilic and it is also clear that the sulfate reducers themselves must be comprised of piezophilic species. Whether or not the fermentative component to the anaerobic community is also pressure-sensitive or piezophilic remains to be investigated. Intriguingly, SR associated with methanotrophy is not strongly affected by pressure. The reasons underlying this observation are not well understood.

Sample decompression and the effects of long-term storage of deep-sea anaerobic samples continue to be formidable obstacles (although surmountable) for deep-sea microbiology and biogeochemistry. As we had no sample material that did not at some point suffer from decompression, we have no accurate baseline assessment of the *in situ* sulfate reducing activity and community in the Japan Trench. Nevertheless, we do observe that samples stored under atmospheric pressure conditions for longer periods can lose their piezophilic character. As such, the experimental quantification of element fluxes and the study of anaerobic processes in deep-sea and subseafloor sediments should take *in situ* pressures into consideration, either by *in situ* experimentation or, at a minimum, immediate re-compression of samples onboard ship.

### Conflict of interest statement

The authors declare that the research was conducted in the absence of any commercial or financial relationships that could be construed as a potential conflict of interest.
